# Three-dimensional culture of endometrial cells from domestic cats: A new *in vitro* platform for assessing plastic toxicity

**DOI:** 10.1371/journal.pone.0217365

**Published:** 2019-05-28

**Authors:** Morgan Dundon, Odile Madden, Pierre Comizzoli

**Affiliations:** 1 Center for Species Survival Smithsonian Conservation Biology Institute, National Zoological Park, Washington, DC, United States of America; 2 Smithsonian Museum Conservation Institute, Suitland, MD, Untied States of America; Harvard Medical School, UNITED STATES

## Abstract

Plastic polymers can be combined with additives that modify physical properties and stability of the material. However, the biocompatibility of those additives is not well known. The objective of the study was to characterize the impact of zinc stearate–a common additive–through the development of a novel three-dimensional (3-D) *in vitro* platform with endometrial cells from domestic cats. Epithelial and stromal cells from adult uteri were isolated and cultured in medium supplemented with 3% Matrigel for two weeks in plastic tissue culture dishes that had been identified as polystyrene with and without zinc stearate by Raman, FTIR, and X-ray fluorescence spectroscopies. Three-dimensional cell structures that were obtained were measured and categorized by shape. Cell viability, proliferation, differentiation, organization, and apoptosis then were assessed by immuno-staining. Results indicated that zinc stearate did not affect 3-D endometrial cell structure morphology, viability, or cellular composition. This first study of a new *in vitro* platform will be useful for studies testing the influence of other additives, drugs, or exogenous hormones.

## Introduction

In recent decades, biomedical research has shifted from glassware to convenient, sterile, disposable plasticware. Plastic supplies are now standard from molecular biology to *in vitro* culture of cells and embryos. The defining components of plastics are polymers, long or net-like organic molecules made of thousands of repeating segments. The vast expanse of these connected, identical segments results in a moldable substance, the properties of which can be tailored to impart flexibility, strength, transparency, or affinity for water or oils, for example [1]. Polymers often are combined with additives that modify the plastic’s working properties during manufacturing or the properties of the finished object. Additives are incorporated at various points in the manufacturing process (i.e., by the compounder, additive manufacturer, or molder) or as contamination, and documentation of their presence often does not reach consumers. Plastics destined for biomedical use also can undergo surface treatments with chemicals, gas plasmas, or electrical discharges to favor certain interactions with biological specimens, like cell attachment or repulsion [2].

While structural and functional properties of tissues is robustly investigated *in vitro*, less is known about the impacts of different conditions, including incubation at physiological temperatures, autoclave, sonication, microwaves, or cryogenic temperatures, on plastic containers. Understanding those potential impacts on plastic properties and integrity of biological samples therefore is critical.

A growing body of research indicates that plastic additives can be bioactive [3,4]. Toxicity of plastics in contact with drinking water and food has been studied extensively [5–7]. Specifically, Bisphenol A and phthalate plasticizers have been shown to leach from plastic and harm gene expression, particularly in reproductive tissues [3, 8–10]. Lubricants like oleamide and erucamide also have been shown to interfere with enzyme activity, ligand-binding, and attachment of organisms to surfaces [4,11,12]. After migrating out of plastic over time, those additives diffuse into aqueous solutions (depending on pH and temperature) or organic solvents [12,13]. This diffusion not only increases cell exposure to the additive but also affects the plastic container’s performance.

Specifically, zinc stearate is a common lubricant added to polystyrene plastics to reduce friction and stickiness during manufacturing. Elemental zinc is involved in many cellular processes including growth and proliferation and has been found to increase survival of vitrified-warmed mice ovaries [14,15]. We hypothesized that lipophilic zinc stearate could interfere with cellular lipid membranes during culture. We also wondered whether highly insoluble zinc stearate would dissociate to some degree and expose cultured cells to zinc ions.

*In vitro* culture models are extensively used for drug and nanomaterial toxicity testing, study of disease, and many more applications [16,17]. Growing cells in three dimensions (3-D) rather than two dimensions can better simulate the *in vivo* cellular environment and encourage natural cell behavior in order to be more organ-specific [18]. Endometrial cells are used in various complex research applications, including cancer studies, which bodes well for more straightforward cytotoxicity studies [17]. However, we know of no plastic toxicity studies on the endometrium or 3-D endometrial cell structures.

Endometrial 3-D cultures could be advantageous in studies about endometrial behavior during different developmental stages in pregnancy, infertility, and specific disorders [19,20]. Maintaining endometrial epithelial cells *in vitro* can be difficult, so developing reliable models for this organ is vital for future studies, and will likely vary between species. Previous studies have developed stable human and mouse endometrial organoid cultures [20,21]. We know of no endometrial 3-D culture studies with domestic cat models even though this species is a unique model for certain biomedical studies and wild felid conservation [22,23]. The objective of the study was to characterize the impact of zinc stearate–a common plastic additive–through the development of a novel three-dimensional *in vitro* platform withendometrial cells from domestic cats.

## Materials and methods

### Plastic characterization and analysis

Two brands of polystyrene tissue culture dishes (35 mm diameter, vacuum-gas plasma treated) were purchased: Falcon brand 353001 dishes from Fisher Scientific (Lot 6101043, Pittsburgh, PA, USA) and CytoOne dishes from USA Scientific (CC7682-3340, Orlando, FL, USA). The Falcon dishes can contain ≤1400 ppm zinc stearate according to the manufacturer. CytoOne dishes were purportedly zinc stearate-free.

Raman spectra of dishes were collected with an NXR FT-Raman module (1064 nm) coupled to a 6700 Fourier transform infrared spectrometer (Thermo Electron Corporation, Madison, WI, USA). Spectra were collected using a 50 μm laser spot and ≤1.3 W laser power. Spectra were co-additions of 256 scans across 100–3701 cm^-1^ (4 cm^-1^ resolution).

Attenuated total reflectance Fourier transform infrared spectroscopy (ATR-FTIR) reference spectra were collected of zinc stearate powder (307564 Zinc Stearate technical grade, Lot #MKBH0073V, Sigma-Aldrich), polystyrene pellets (Cat #039A Polystyrene, nominal M.W. 2–300,000, Scientific Polymer Products, Inc., Ontario, New York, USA), and dish fragments with a Thermo Nicolet 6700 FTIR spectrometer with Golden Gate single-bounce ATR accessory and DTGS detector. Spectra were a co-addition of 64 scans collected at 4 cm^-1^ resolution and were ATR corrected.

Dishes were screened for the presence of zinc with a Bruker Artax 400 μXRF spectrometer equipped with a rhodium tube, poly-capillary lens with ~100 μm focal spot and a Peltier-cooled silicon drift detector. Spectra were collected at 50 kV and 480μA for 240 seconds under a helium flush. The instrument detects elements heavier than silicon, so polystyrene does not contribute to the spectrum.

The presence of zinc stearate, as opposed to other zinc compounds, was confirmed in triplicate by solvent extraction. Culture dish fragments (0.75 grams) were weighed and transferred to 15 mL glass centrifuge tubes with 9 mL tetrahydrofuran (THF) (CAS 109-99-9, Fisher Scientific T425-1) (16:1 solvent-to-mass ratio). Vials sat undisturbed at room temperature until the polystyrene dissolved completely in the solvent. Within twelve hours the polystyrene dissolved into two visible layers, one more viscous than the other. Thirty-one days later the plastic dissolved completely into a uniform solution, with a small quantity of white powder at the bottom of the vial. The supernatant was decanted with glass Pasteur pipettes. The residual material was washed with 3 mL fresh THF, decanted again, and dried overnight. Solid residue was collected with a metal spatula and identified by FTIR as polystyrene and zinc stearate.

### Collection/Isolation of endometrial cells

The study did not require the approval of the Animal Care and Use Committee of the Smithsonian Institution because reproductive tracts were collected at local veterinary clinics as byproducts from owner-requested routine ovariohysterectomies. Tissues from adult domestic cats (age 8 months or older, after first estrus cycle) were placed immediately in phosphate-buffered saline (PBS; 4°C) supplemented with 100 IU/ml penicillin and 100 μg/ml streptomycin (Sigma-Aldrich) and transported to the laboratory. Uterine horns (3–5 adult uteri per replicate) were isolated, cut into ~1 cm sections, placed in 1% trypsin (Sigma-Aldrich) in PBS, rinsed twice by inserting the pipette tip into the lumen, and incubated at 4° C for 1 hour and at room temperature for 45 min (~22°C). Sections were then placed in Dulbecco’s Modified Eagle Medium/Nutrient Mixture F-12 (DMEM/F-12) (Life Technologies) supplemented with 100 μg/mL penicillin/streptomycin (Sigma-Aldrich), 1 mM HEPES buffer, and 1 μL/mL Amphotericin B (Gibco) (basal medium), supplemented with 10% fetal calf serum (FCS) (Invitrogen) to stop trypsination. Cell clumps were collected by scraping with the blunt end of a scalpel blade. Cell clumps were washed gently with PBS twice and transferred to fresh basal medium supplemented with 10% FCS and aspirated gently ~100X to separate cell clumps. All cells collected on a given day (3–5 uteri) were pooled to account for variability in estrus cycle among cats.

### 3-D culture

Equal volumes of cell mixture (300 μL) were placed into the 35 mm polystyrene dishes, diluted to 1 mL with basal medium supplemented with 10% FCS, and placed in a humidified incubator at 38.5°C and 5% CO_2_ for 48 hours. 48 hours after plating, the culture medium was removed, cells were washed with pre-warmed PBS, and incubated in 1 mL of 3-D culture medium (basal medium supplemented with 5 ng/mL epidermal growth factor (EGF) (Gibco), a 1:100 dilution of insulin-transferrin selenium (ITS) (Gibco), and 3% Matrigel (Corning) [17]. Medium was replaced every 36–48 hours until Day 14 when bioassays were performed. Pilot studies were performed under the same conditions from 1 to 4 weeks before 2 weeks was chosen as the most consistent culture period.

### Morphological assessment of 3-D endometrial cell structures

**Fig 1:**
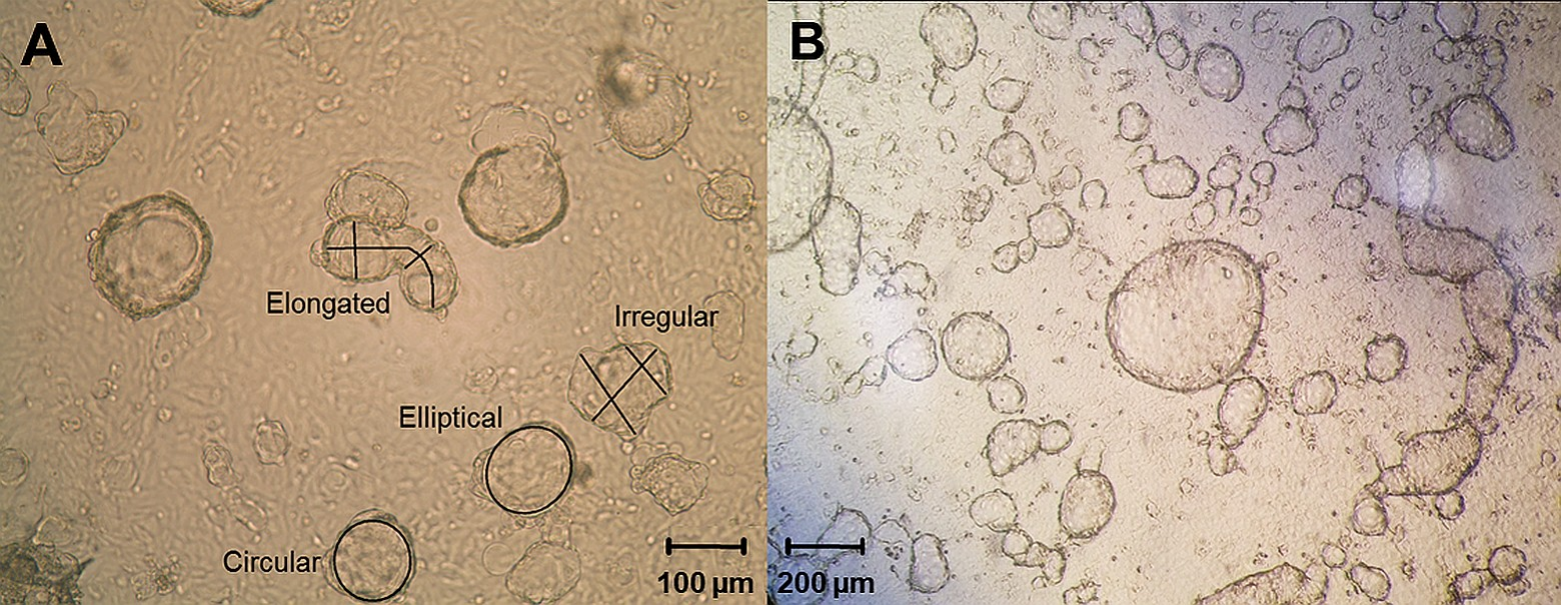
Examples of 3-D endometrial cell structure categories after two weeks of *in vitro* culture. A) Each shape classification is labeled with its classification by visual inspection. Black lines and circles represent measurements taken using SPOT Advanced Software 3.5.9 with the measurement tools. Photo area: 1.05 mm^2^. B) Lower magnification after two weeks in culture.

Three photographs were taken of each dish at 100X magnification with an Olympus BX41 light microscope and SPOT advanced software 3.5.9 (Diagnostic Instruments Inc.). Observed 3-D endometrial cell structures were categorized visually as circular, elliptical, elongated, or irregular (Fig 1) and counted as a percentage per photograph. The length and width of each structure was measured with SPOT software calibrated for each microscope magnification. The diameter or major/minor axes of circular and elliptical structures were measured with the circle and ellipse tools. For elongated structures, length was measured manually with the curve tool, and widths with straight drawn lines taken at two points. Irregular structures were measured by lengths and widths at two points with the line tool (Fig 1A). Measured dimensions for the lengths and widths as well as the percent occurrence were pooled for all 3-D endometrial cell structures within each shape category. Both data sets were analyzed with a Kruskal-Wallis test and plotted as the median with interquartile range (IQR) to visualize the data spread.

### Assessment of cellular viability

Endometrial cell viability was evaluated using a LiveDead Viability/Cytotoxicity kit (Life Technologies) according to manufacturer’s instructions. Briefly, 3-D endometrial cell structures were washed with PBS, incubated with 2 μM calcein AM and 4 μM Ethidium homodimer-1 (EthD-1) in PBS for 45 minutes at room temperature in the dark, washed twice, mounted directly in the dish under coverslips with Vectashield (Vector Laboratories), sealed with nail polish, and observed at 518 nm and 593 nm with an Olympus BX41 microscope equipped with epifluorescence. Three photos were taken per dish at 200X magnification. The percentage of live cells were calculated by dividing the number of live cells by the total number of cells counted with the Multi-point tool in ImageJ (National Institutes of Health).

### Assessment of DNA synthesis and proliferation

DNA synthesis was assessed with Click-iT Alexa Fluor 488 Imaging Kit protocol (Molecular Probes). Briefly, 3-D endometrial cell structures were incubated in 10 μM 5-ethynyl-2'-deoxyuridine (EdU) (Molecular Probes) overnight at 38.5°C to allow the nucleoside analog of thymidine to incorporate into DNA. Cells were then fixed with 4% paraformaldehyde, permeabilized with 0.5% Triton X-100, stained, counterstained with Hoechst, washed twice, mounted directly in the dish with Vectashield (Vector Laboratories) under coverslips, and sealed with nail polish. Three photos were taken at 200X magnification per culture dish. EdU-positive nuclei were scored by dividing the Edu-positive nuclei by the total number of nuclei (visualized by Hoechst staining). Scores were expressed as a percentage of EdU-positive nuclei, or proliferating cells counted with ImageJ. A negative control was performed by staining cells that had not been incubated with 10 μM EdU.

### Immunostaining of 3-D endometrial cell structures

For all experiments, 3-D endometrial cell structures were washed in PBS, fixed in 4% paraformaldehyde for 5 minutes at ambient temperature, permeabilized in 0.5% Triton X-100 for 10 min, blocked in 3% BSA in PBS for 30 min, incubated in primary antibody in 1% BSA in PBS overnight at 4°C, washed twice with PBS, incubated in secondary antibody in 1% BSA in PBS for 1 hour in the dark, washed twice, incubated in primary antibody in 1% BSA in PBS overnight at 4C, washed twice in PBS, and incubated in secondary antibodies at room temperature in the dark for 1.5 hours, washed twice, counterstained with (Hoechst, 1:2000) (Fisher), mounted directly in the culture dish with Vectashield (Vector Laboratories), and sealed with nail polish for observation. Negative controls were performed by staining cells that had not been incubated with the primary antibodies. For stromal cell staining, primary antibody was anti-vimentin (1:200) (Abcam) and secondary was FITC anti-mouse (1:100) (Fisher). For epithelial cells, primary antibody was anti-cytokeratin (1:200) (Abcam) and secondary was Texas Red anti-rabbit (1:100) (Fisher). Percentages of epithelial cells were scored by dividing the number of cytokeratin-stained cells by the total number of cells (total cytokeratin and vimentin-stained cells) counted with ImageJ. Three photos were taken per dish at 200X magnification. Scores were expressed as a percentage of epithelial cells. For structure polarity, the primary antibody was anti-laminin (Abcam) and rhodamine phalloidin, (1:500) (Life Technologies) and the secondary antibody was FITC anti-rabbit 1:100 (Fisher).

### Immunostaining of uterine horns

Uterine horn tissue samples were embedded in paraffin, sectioned at 5 μm thickness, mounted onto Fisher Superfrost Excell microscope slides, and stored at 4°C. Sections were dewaxed with xylene and ethanol prior to retrieving antigens with citric acid/EDTA buffer in a 95°C water bath, blocked with 5% BSA and 0.5% Triton X-100, incubated in primary antibodies (anti-cytokeratin 1:200, anti-vimentin 1:200 or rhodamine phalloidin 1:500, anti-laminin 1:500) in 5% BSA overnight at 4°C, washed, incubated in secondary antibodies (anti-rabbit Texas Red 1:100, anti-mouse FITC 1:100 or anti-rabbit FITC 1:100) in PBS for 1 hour, washed, counterstained with Hoechst (1:2000) in PBS for 10 min, washed, mounted with Vectashield (Vector Laboratories), and sealed with nail polish for observation. A negative control was incubated without primary antibodies prior to incubation with secondary antibodies.

### Assessment of apoptosis

Approximately 30 3-D endometrial cell structures of varying shape were aspirated from one culture dish per treatment group and stained with Annexin V conjugated with FITC and propidium iodide as per Annexin V-FITC Apoptosis Detection Kit protocol (EMD Millipore), then counterstained with Hoechst in a 1:2000 dilution in PBS for 30 mins at room temp in the dark, mounted on glass sides with Vectashield (Vector Laboratories) and coverslipped for fluorescence microscopy. A negative control was performed by incubating cells in binding buffer without Annexin V and propidium iodide and then counterstaining with Hoechst. A positive control was performed by heating cells at 55°C for 20 min prior to staining. Three photos were taken per dish at 200X magnification. Early apoptotic cells were scored by dividing the Annexin V-stained cells by the total number of nuclei (visualized by Hoechst staining) counted with ImageJ. Scores were expressed as a percentage of early apoptotic cells.

### Experimental design

After a pilot study verifying culture conditions that would lead to predictable cell morphologies and growth, the impact of zinc stearate-containing polystyrene was evaluated by culturing feline endometrial cells in three conditions: polystyrene dishes that did not contain zinc stearate (control, NZ), polystyrene dishes that contained zinc stearate (low concentration condition, Z), and pure zinc stearate in a NZ dish (high concentration condition, NZ+; S1 Fig).

The composition of the dishes was determined with Raman, FTIR, and μXRF spectroscopies. Dishes were then divided into two groups: with and without zinc stearate. It was decided that a 3-D culture would allow longer cell exposure without passaging and transferring the cells to new dishes. A 2-week long culture period was used after a preliminary study of cell culture performance from 1–4 weeks under the described conditions. 5–19 cultures (replicates) were performed for each bioassay, which corresponds to 11–16 dishes assessed for each treatment group within each bioassay.

To evaluate the effect of zinc stearate on cells without the constraint of the polystyrene, a third treatment group with pure zinc stearate powder was added. It was quickly discovered that zinc stearate powder floats, preventing cell contact and making it impossible to change the culture medium without removing the powder too. Instead, 1–2 mg of the zinc stearate reference powder was melted at 130° C onto 5 mm round glass coverslips (Electron Microscopy Sciences). One coated coverslip was inserted along the edge of NZ culture dish, beneath the culture medium for the duration of the cell culture (S1 Fig). This setup anchored the zinc stearate and facilitated contact with the endometrial cells. 1–2 mg of zinc stearate fit on a small 5 mm coverslip, which left most of the dish surface open to cell attachment. Assuming approximately 1 gram of cell tissue and culture medium in each dish, this dose fit in the published range of LD_50_ for zinc stearate in mice (354 mg/kg for intraperitoneal exposure; >10,000 mg/kg for ingestion) [24].

Groups of cultures were initiated over many weeks, and coincided with delivery of fresh cat uteri by local veterinarians. On a given delivery day (one replicate), endometrial cells were harvested by scraping. A portion of the cells harvested that day were divided among Z, NZ, and NZ+ tissue culture dishes and grown for fourteen days. On Day 14, cultured 3-D endometrial cell structures were photographed, categorized by shape, measured, and counted. Bioassays for viability, DNA synthesis (evidence of proliferation), cell type, cell polarity, and apoptosis were performed. Within each replicate, the number of treatment groups tested (at least two) and assays performed (1 or 2) varied with the number of available uteri that day. All assay results within a replicate (3–5 uteri per replicate; n = 5–19 replicates) were pooled and reported as median values with interquartile range (IQR) to visualize the data spread.

### Statistical analysis

Data from all replicates were pooled to determine the percentages of viable, proliferating, and epithelial cells for each treatment group, and analyzed using a nonparametric Kruskal-Wallis test to compare the median and IQR of each treatment group. Apoptosis detection percentages were pooled from all replicates and analyzed with a one-way ANOVA.

## Results

### Materials characterization

**Fig 2:**
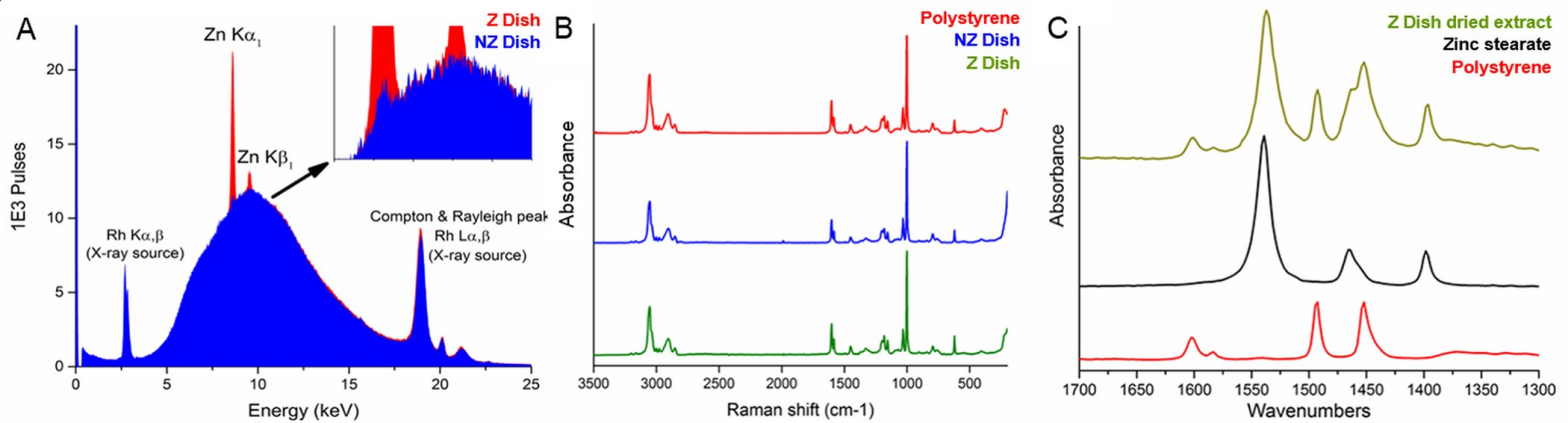
Materials characterization of plastic culture dishes. A) X-Ray fluorescence comparison of zinc presence between non-Zinc dishes (blue) and Zinc dishes (red). B) FT-Raman comparison of a polystyrene reference spectrum (top/red) with a non-Zinc dish (center/blue) and Zinc dish (bottom/green). Intact culture dishes were made of polystyrene but did not reveal any other noticeable structural differences. C) FTIR spectra of the Zinc dish dried extract (top/green), zinc stearate powder (center/black) and polystyrene reference (bottom/red).

Using Raman and FTIR spectroscopies, all culture dishes were confirmed to be polystyrene plastics. μXRF spectrometry revealed a clear difference in the intensity of zinc Kα_1_ and Kβ_1_ peaks (8.64 and 9.57 keV) between the Z and NZ dishes (Fig 2A). A very small Kα_1_ peak was visible in spectra of the NZ dishes as well, which should have no zinc present, but was negligible compared to the Z dishes. Zinc stearate was confirmed as the source of zinc by solvent extraction and FTIR. The spectrum of the dried extract (Fig 2C) showed zinc stearate peaks at 1539, 1466 (shoulder), and 1398 cm^-1^ that were distinct from the residual polystyrene signature.

### Development of the 3D-culture system

**Fig 3:**
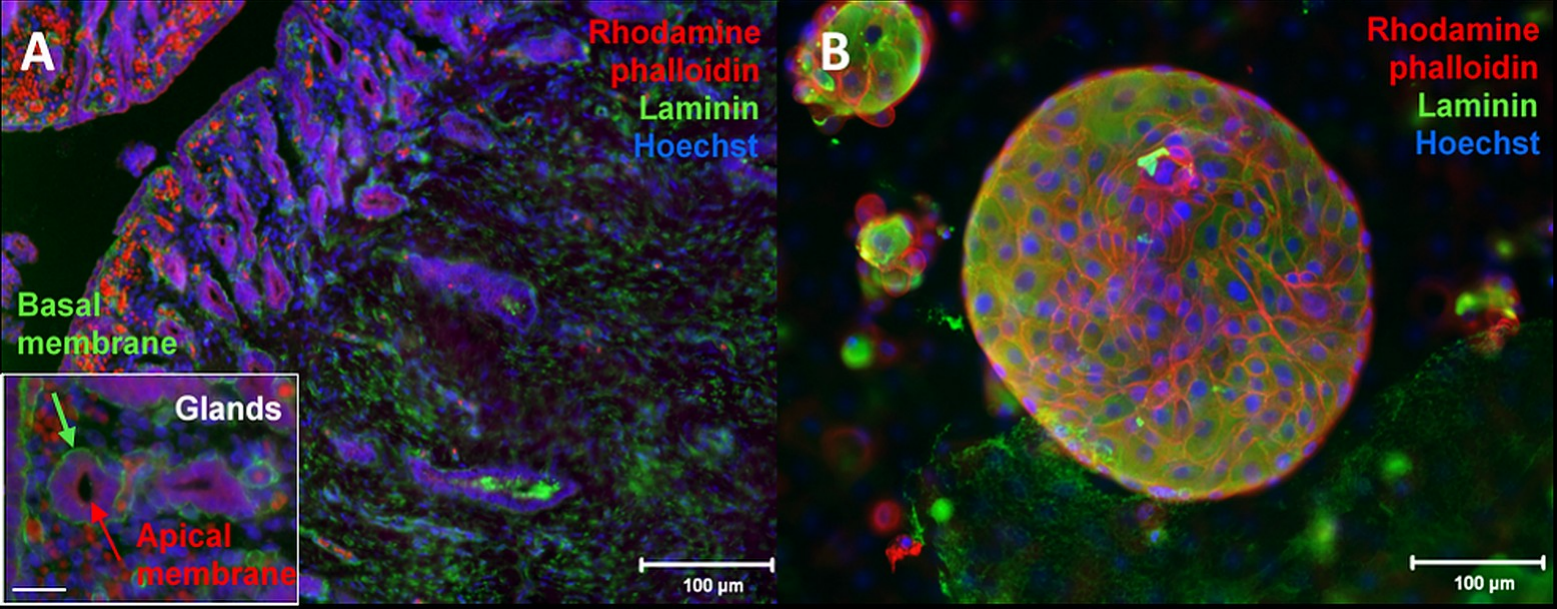
Immunostaining for cell polarity. Fluorescent micrograph of immunostaining with Rhodamine phalloidin (cytoskeleton = red), laminin (basement membrane = green), and Hoechst (nuclei = blue) (A) 5 μm uterine horn cross-section. Inset: 400X micrograph of two individual glandular structures which show apico-basal polarity where the laminin-rich basement membrane faces away from the lumen. Bar = 50 μm (B) Circular cell structure stained after two weeks of endometrial cell culture.

The pilot study showed that 1 week of culture was not long enough to obtain fully formed 3-D endometrial cell structures. However, 3- and 4-week cultures showed that cells attached to the dish were still viable, but the suspended 3-D cell structures, while shown to be proliferating, were beginning to detach from the dish at this point of the culture. A 2-week culture therefore was found to be optimal within a single culture dish to grow the 3-D endometrial cell structures consistently. After two weeks of culture, 3-D cell structures were grouped by shape into four categories: circular, elliptical, elongated, and irregular (Fig 1). Control cross-sections of uterine horns were stained with rhodamine phalloidin and laminin to show glandular structures with apical and basal poles (Fig 3A). Structures obtained after two weeks of culture were stained the same way. Rhodamine phalloidin surrounded individual cells and laminin was observed to coat the outer surfaces (Fig 3B). However, epifluorescence observations were made on the whole outer surface of the cell structures and not on a cross-section (like for the uterine horns).

### Influence of zinc stearate on cell morphology, viability, proliferation, differentiation, and apoptosis

**Fig 4:**
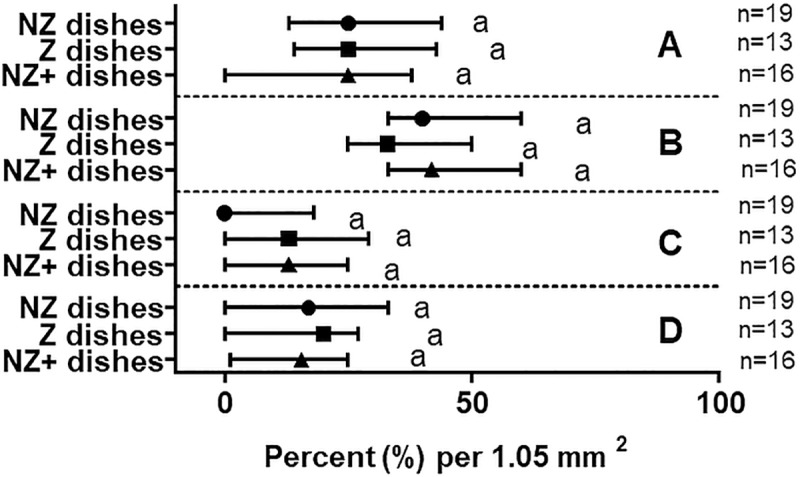
Shape classification and occurrence of 3-D endometrial cell structures. Occurrence as a percentage per micrograph area of circular (A), elliptical (B), elongated (C), or irregular shapes (D) observed after two weeks of culture in different treatment groups (Median with IQR). Number of replicates for each treatment group are in parentheses. Matching lowercase letters indicate the absence of difference within each shape (P>0.05).

**Fig 5:**
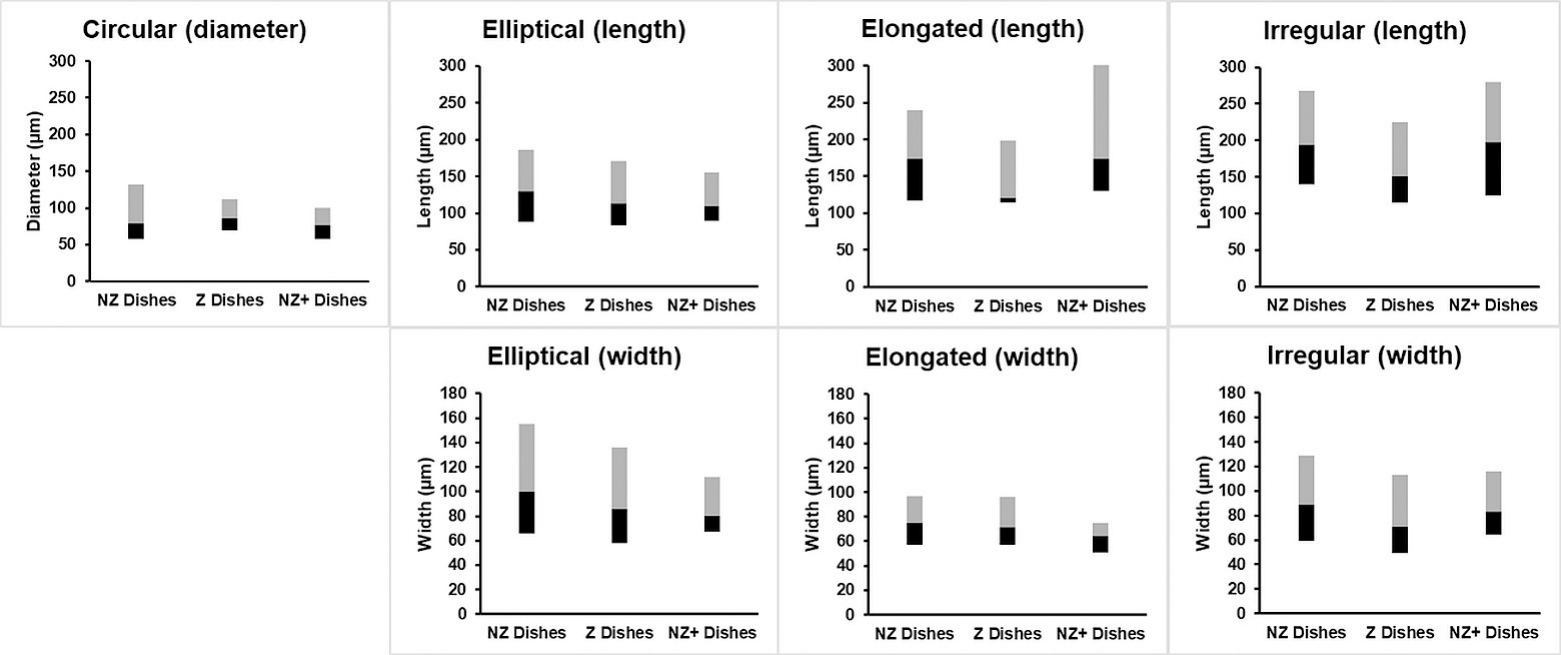
Morphological measurements of 3-D endometrial cell structures. Diameter, lengths, and widths of structures across all observed shapes and treatment groups after two weeks of culture (Median with IQR). The black bar represents quartile 1 (25^th^ percentile) to the median (50^th^ percentile), and the gray bar represents the median to quartile 3 (75^th^ percentile). Number of replicates for each treatment group are listed under each plot. Matching lowercase letters indicate the absence of statistical difference within each category (P>0.05).

The median occurrence of each shape did not vary among treatment groups (P>0.05), which were 23–25% (circular), 33–42% (elliptical), 0–13% (elongated), and 15–20% (irregular). The elliptical shape occurred slightly more often but the difference was not statistically significant (P>0.05; Fig 4). Measured dimensions did not vary (median with IQR) among treatment groups within each shape category as well (P>0.05) (Fig 5).

**Fig 6:**
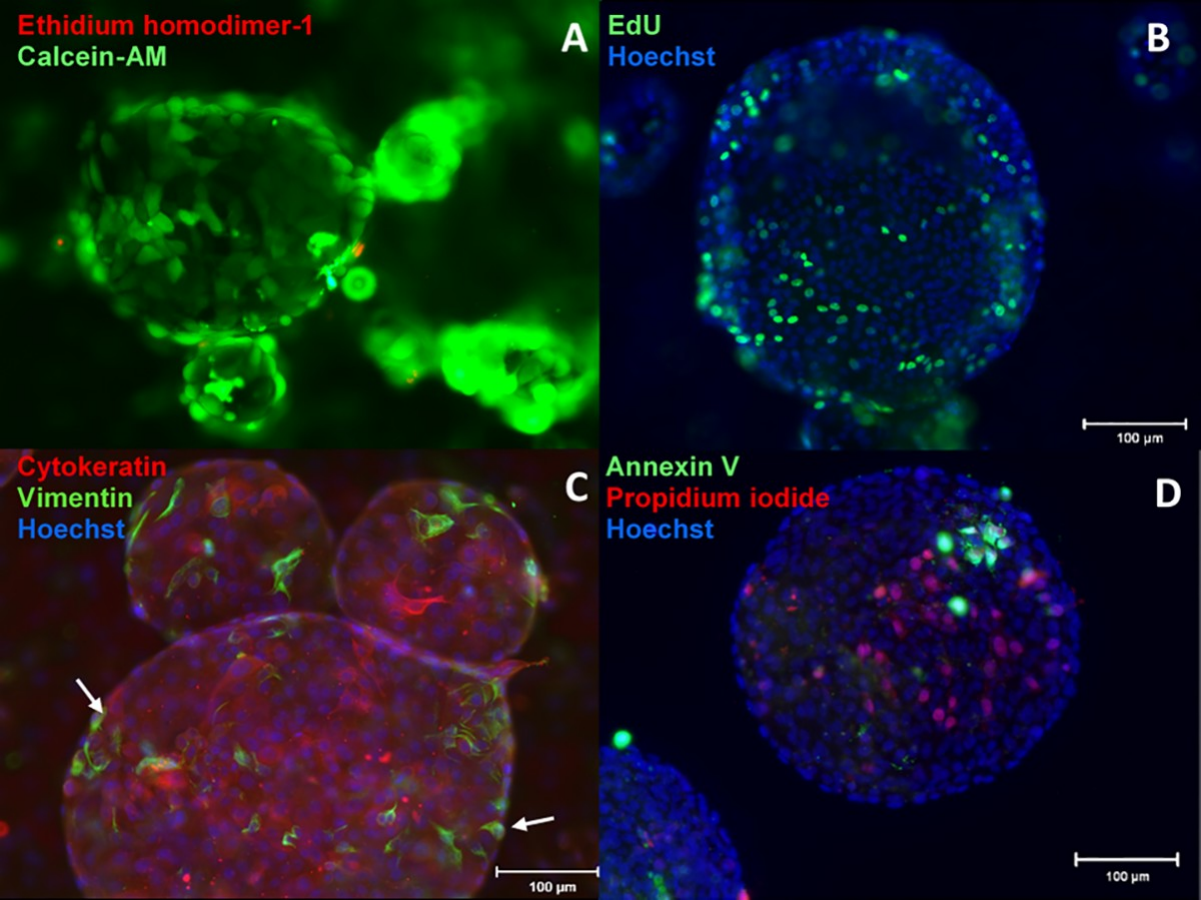
Fluorescent micrographs of 3-D endometrial cell structures after 2 weeks of culture. (cell nuclei are counterstained with Hoechst, blue). A) Viability assay with Calcein-AM (live cells) and ethidium homodimer-1 (dead cells) show hollow, lumen-like structures. B) Proliferation assay with EdU showing cells actively synthesizing DNA and proliferating within a spherical cell structure. C) Staining of epithelial (cytokeratin) and stromal cells (vimentin) showing primarily epithelial cells forming the 3-D endometrial cell structures with stromal cells (white arrow) lining the outer surface. D) Detection of early apoptotic nuclei (annexin V) and dead cells (propidium iodide).

**Fig 7:**
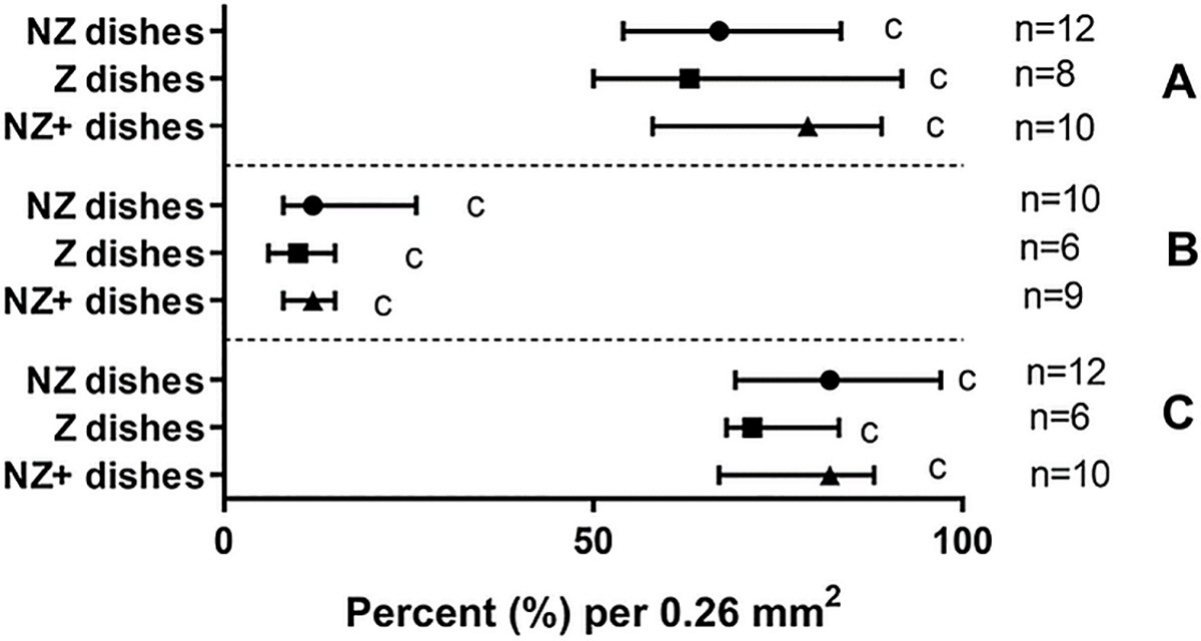
Viability, proliferation, and differentiation bioassay of 3-D endometrial cell structures. Percentages of viable cells (A), proliferating cells (B) or cytokeratin-rich epithelial cells (C) after two weeks of culture in different treatment groups (Median with IQR). Number of replicates for each treatment group are in parentheses. Matching lowercase letters indicate the absence of difference within each shape (P>0.05).

**Fig 8:**
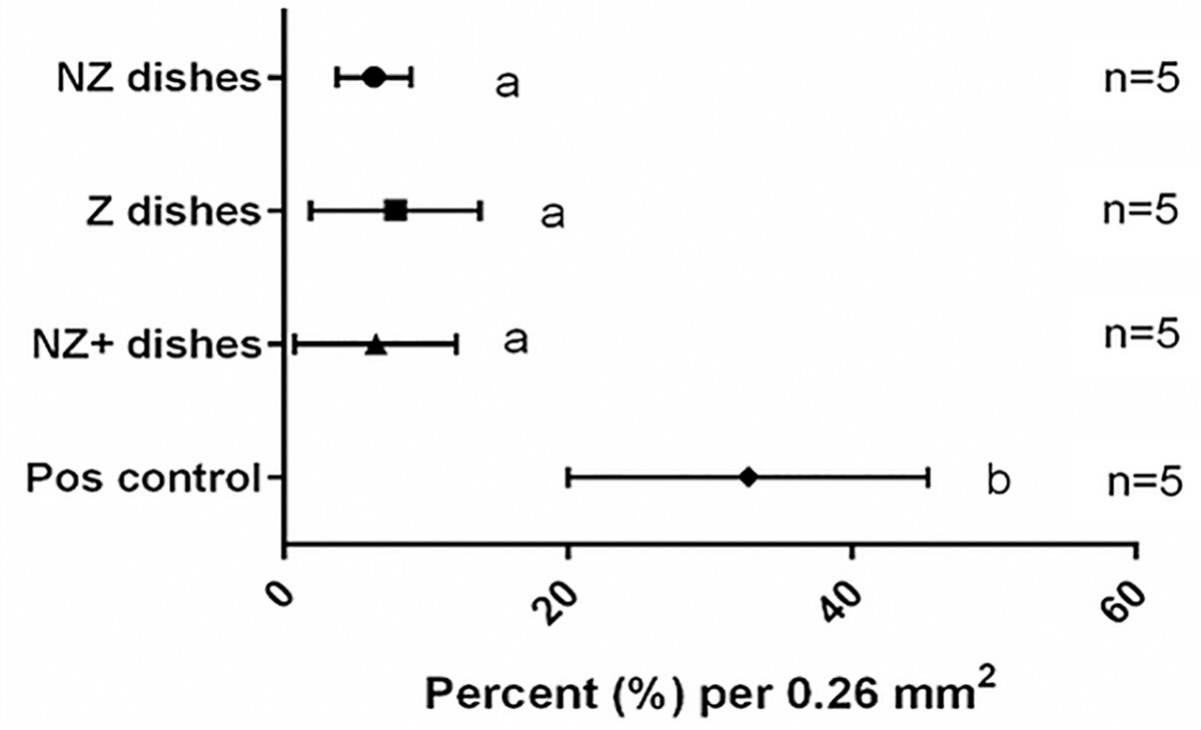
Early apoptosis assay results. Percentage of early apoptotic cells after two week of culture in different treatment groups (Mean ± SD). Number of replicates for each treatment group are in parentheses. Matching lowercase letters indicate the absence of statistical difference between treatments (P>0.05). Positive control group showed significant difference (P = 5 × 10^−13^).

Examples of the staining performed in Fig 6 show 3-D endometrial cell structures stained for viability, proliferation, differentiation, and apoptosis (Fig 6A–6D). No differences were observed among treatment groups in terms of cell viability, proliferation, relative proportions of epithelial and stromal cells, or proportion of apoptotic cells (Figs 7 and 8). Median percentages of cellular viability were 71% (NZ dishes), 68% (Z dishes), and 74% (NZ+ dishes) and showed no significant difference in the median among treatment groups (P>0.05) (Fig 7A). The percentages of proliferating cells did not differ among groups either (P>0.05): 13% (NZ dishes), 17% (Z dishes), and 12% (NZ+ dishes) (Fig 7B). Similarly, cellular compositions (epithelial vs. stromal cells) were not found to be statistically different among groups (P>0.05) at 81% (NZ dishes), 72% (Z dishes) and 82% (NZ+ dishes) (Fig 7C). Lastly, the percentage of early apoptotic cells was not different among treatment groups (P>0.05) at 6% (NZ dishes), 9% (Z dishes), and 6% (NA+ dishes) (Fig 8). Variation in the NZ dishes for the apoptosis assay was 54–56% lower than the NZ+ and Z dishes (Fig 8).

## Discussion

After a thorough plastic and additive characterization, we developed a preliminary 3-D *in vitro* culture platform for studying the effects of plastic on the endometrial cells. 3-D endometrial cell structures grown from epithelial and stromal cells were viable and proliferating. Structural properties of these structures were similar to endometrial organizations found in cat uterine horns. Although no significant effects from zinc stearate were observed during the two-week culture, this platform could be used, with further study and validation, for future studies investigating the influence of other plastic additives, drugs, or exogenous hormones.

Zinc stearate, a common plastic processing aid, was detected in some dishes, which agreed with information provided by the tissue dish manufacturer, but its presence was inconsistent between brands and batches. The presence of unreported additives in plastics is commonplace. Hence, characterization of plastic labware prior to use with cell cultures is good practice. An analytical protocol for identifying the components of a plastic with non-destructive spectroscopic methods and solvent extraction was described. Other studies have used water and extraction buffers over long periods to extract plastic leachates, from days to months [7,25]. Indeed, lengthy extractions may be necessary to identify some organic compounds, like the stearate in this study. Here, the overwhelming FTIR signal of the polymer necessitated extraction of the low concentration additive for independent analysis. Another method suited to identification of ppm and ppb concentrations of plastic additives is gas chromatography/mass spectroscopy, particularly with pyrolysis. While we aimed merely to identify the target additive, quantification was also possible.

Zinc stearate’s aqueous insolubility and low density necessitated a creative presentation in the NZ+ dishes. Ultimately, melting the compound onto small glass coverslips served to hold the compound together and weigh it down in the dishes. Temperature was carefully controlled so as to melt but not decompose the stearate. Plastic additives that are soluble in aqueous media would be simpler to implement in the proposed model as concentration and exposure can be closely controlled. A future study on the solubility of zinc stearate in polystyrene at differing pH levels in culture media, similar to Jenke 2010, may give insight into its solubility and leaching behavior [25].

Previous cytotoxicity studies have used contact exposure to detect leachates from plastic materials, particularly in food contact and pharmaceutical studies [7,26,27]. In this project, cells were exposed to zinc stearate as a solid, pure pellet, and in the context of a compounded plastic. Culturing in zinc-containing and zinc-free polystyrene dishes was convenient and served the valuable purpose of assessing bioavailability. The act of compounding an additive into a plastic also sequesters that compound, making it less bioavailable. Plastic lubricants reduce friction by forming a fine layer on the plastic surface, which is one potential pathway for toxic exposure. Contact with aqueous solutions that leach compounds back out of the plastic is another potential exposure route. By presenting zinc stearate to the cells in pure and compounded form, we could assess toxicity of the compound and its exposure potential in the plastic. In this study, we wanted to ensure the culture dishes containing zinc stearate (Z Dishes) were exposed to cells for as long as possible to initiate leaching, so no passaging was performed. However, in future studies, longer culturing times would help to examine longer-term effects. This would be particularly useful for the zinc tab treatment group (NZ+ Dishes), where tabs could be transferred to new culture dishes after passaging very easily.

During the pilot study, we found that the cell mixture would grow into a more spherical shape and others more elongated after 1 week of culture. Endometrial 3-D cultures performed in other species also observe round structures similar to those seen here, although different media formulations were used [20,21]. Boretto et al. suggest using an endometrium-specific matrix in place of Matrigel in future studies, which could help optimize these *in vitro* models [21]. Increased variation in 3-D cell structure morphologies seen in this study could also be due to the number of adherent cells present in the individual culture dishes. Since the presence of zinc stearate did not affect these observed shapes, it is possible that larger numbers of adherent cells may cause spheroid shapes to join together to make more irregular shapes.

Cells were grown in 3-D culture but morphologies of the cell structures were reported from surface measurements only. Since no significant difference in morphologies were observed from the surface measurements, we did not expect that the volumes of the 3-D cell structures would show any statistically significant difference as well. In future studies done with a different test compound that does have a negative impact *in vitro*, 3-D measurements of morphology (e.g. confocal microscopy) will be necessary. From the observation of the surface (Figs 3 and 6), 3-D endometrial ecell structures obtained after 2 weeks of culture had shapes and compositions comparable to observed endometrial 3-D structures from other species [17,20].

Clear differentiation between cytokeratin-rich epithelial cells and vimentin-stained stromal cells showed the 3-D structures consisted primarily of epithelial cells with stromal cells lining the perimeter, consist with previous studies, however confocal microscopy will be necessary in future work to determine how these cells organize throughout the entire 3-D endometrial cell structure [28]. Percentages of proliferating cells were lower than those described in examples of endometrial culture and were scattered across the 3-D endometrial cell structures rather than being concentrated in particular areas (Figs 6B and 7B). However, with longer culture periods in this study (14 days) compared to eight days seen previously, proliferation may decrease over time without passaging cells, but was not halted completely in this study [17].

The lack of clear apicobasal polarity observed after the completion of the two-week culture could be because visualizing a 3-D cell structure by the surface without confocal microscopy made comparing polarity difficult without observing the cross-section of the 3-D cell structures (Fig 3). Histological sections were technically difficult to prepare due to the small size and fragile nature of the 3-D cell structures. Assessing correct cell polarity formation with confocal microscopy will be important in future studies to be able to mirror the *in vivo* environment as accurately as possible. Secondly, the observed polarity could be due to a mixture of cell types being grown in tissue culture-treated dishes without permeable supports which aid in culturing polarized cells [29]. In this study, we did not want to introduce another material (such as permeable supports) into our culture dishes in order to minimize possible influence the new material may have had on our assay results. Other polarity markers such as E-cadherin, claudins, and ZO-1 could be used in future studies as well to observe adherent and tight junctions between cells along with rhodamine phalloidin and laminin to further characterize the cell organization within these 3-D endometrial cell structures [17]. This combination of instrumentation and markers would help to confirm if glandular structures with hollow lumen formation are being grown.

Analysis of the epithelial-stromal co-culture after 2 weeks show high viability rates, which indicates that co-culture of epithelial and stromal cells could survive in single dishes without passaging during that time period. However, for longer culture times and toxicity studies with chronic exposure, passaging would be required to create long-term stability, as shown in previous studies [19,21]. Given most plastics are not obviously or acutely toxic, this may allow detection of more subtle impacts that occur with long exposure to a compound.

Apoptosis is a common indicator that has been used in drug screening tests and in studies to understand lumen formation in glandular epithelium [18,30,31]. However, more in-depth markers could be used in future studies to better show possible induced toxicity since cultures were not tested for mycoplasma before Annexin V analysis. The lower percentage of apoptosis observed in the treatment groups compared to the heated cells in the positive suggests no impact of the polystyrene, but does not explain what induced apoptosis other than possibly a homeostatic mechanism typically seen in cell populations in tissues [31].

Depending on the additive being studied, the bioassays described could be supplemented with other targeted tests. Assays for gene expression or epigenetic changes would be a logical next step. These assays could also be used to compare the endometrial 3-D culture to gene expression seen *in vivo* [20]. If plastic leachates show changes in viability, proliferation, differentiation, and also gene expression, then the mechanism for the toxicity or other interference could begin to be established, which is an area of research that is lacking, particularly for endocrine disrupting plastic leachates [32].

Techniques described here could be used in future studies to assess the impact of plastics in contact with viable biomaterials for longer periods of time, such as those used for cryopreservation of reproductive tissues. The use of these various containers at ultra-cold temperatures has the potential to influence the material properties of the plastic during freezing and thawing. For example, PVC straws can become more brittle and break after freezing with liquid nitrogen [33]. These possible changes in material properties of containers during freezing and thawing need to be considered for long-term biomaterial storage. Further studies are needed to determine whether stresses plastic containers sustain during long-term culture and frozen storage could result in leaching of plastic additives and negatively impact viability of cultures and stored tissues. This is particularly relevant for chronic, long-term exposure, which is lacking in most *in vitro* models [18]. To truly assess the performance of plastics for culture and tissue storage, a variety of plastic leachates should be tested rather than only a single compound, which may help to identify previously unknown toxicants, like endocrine disruptors [32].

After a thorough material characterization, this research shows promise towards the development of a successful 3-D *in vitro* cell culture platform for studying the plastic toxicity. 3-D cell structures grown from uterine epithelial and stromal cells formed viable and proliferating 3-D structures having structures comparable to endometrium, although more validation is necessary. Although no significant effects from zinc stearate were observed during the two-week culture, this *in vitro* platform could be used for future studies testing effects of other plastic additives, drugs, or exogenous hormones after further validation.

## Supporting information

S1 Fig NZ+ dishes setup. Left) Schematic of the NZ+ dishes with the inserted coverslip covered with melted zinc stearate powder. Right) Aerial photo of a coverslip with zinc stearate powder along the edge of a culture dish.
(TIF)

S1 Dataset Bioassay results data. (XLSX)

S2 Dataset Morphology measurements data. (XLSX)
