# Supplementary figures and images for: Three-dimensional culture of endometrial cells from domestic cats: A new in vitro platform for assessing plastic toxicity
Source: PLoS One. 2019 May 28;14(5):e0217365. doi: 10.1371/journal.pone.0217365 (PMC6538153; doi:10.1371/journal.pone.0217365)

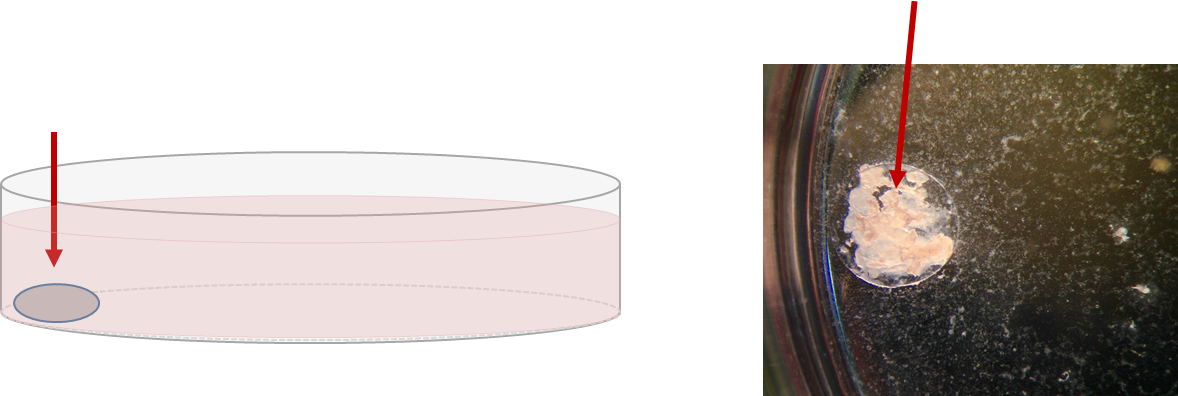

Supplement: S1 Fig — Left) Schematic of the NZ+ dishes with the inserted coverslip covered with melted zinc stearate powder. Right) Aerial photo of a coverslip with zinc stearate powder along the edge of a culture dish. (TIF) [file pone.0217365.s001.tif]
